# Association between circadian rhythm disruption and the risk of malignancy in patients with thyroid nodules: a propensity score-matched study

**DOI:** 10.3389/fendo.2026.1832788

**Published:** 2026-05-12

**Authors:** Pengfei Gu, Beibei Zhang, Ziliang Ding, Yukun Wei, Yahui Sun, Pengzhao Song, Weiwei Zou, Yong Han, Fengli Guo

**Affiliations:** 1 Department of Thyroid Surgery, Binzhou Medical University Hospital, Binzhou, Shandong, China; 2 Medical Integration and Practice Center, Shandong University, Jinan, Shandong, China; 3 Department of Radiology, Binzhou People’s Hospital Affiliated to Shandong First Medical University, Binzhou, Shandong, China; 4 Department of Breast Surgery, Binzhou Medical University Hospital, Binzhou, Shandong, China

**Keywords:** circadian rhythm disruption, propensity score matching, risk factor, thyroid cancer, thyroid nodule

## Abstract

**Background:**

The rising incidence of thyroid cancer (TC) underscores the need to identify modifiable risk factors for malignancy in patients with thyroid nodules. The role of circadian rhythm disruption (CRD), a pervasive modern lifestyle factor, in TC is poorly understood. We aimed to investigate the association between cumulative CRD and the risk of malignancy and aggressiveness in patients with thyroid nodules.

**Methods:**

We conducted a hospital-based, propensity score-matched study involving 2,541 patients who underwent thyroidectomy for thyroid nodules between 2016 and 2019. A novel cumulative circadian rhythm disruption index (cCRDI), integrating sleep insufficiency, shift work history, chronotype/mid-sleep timing, and dietary irregularity, was calculated from preoperative questionnaires. The primary outcome was the definitive postoperative histopathological diagnosis (benign vs. malignant). Propensity score matching (PSM) (1:1) was performed to balance baseline confounders (e.g., age, sex, BMI, TSH levels, and autoimmune status), yielding a cohort of 850 matched pairs (1,700 patients). Conditional logistic regression was used to assess the association between cCRDI and malignancy.

**Results:**

In the matched cohort, a strong, dose-dependent relationship was observed. Compared to the No CRD group, the adjusted odds ratios (aOR) for malignancy were 1.58 (95% CI: 1.15–2.15) for Low CRD, 1.85 (95% CI: 1.30–2.62) for Moderate CRD, and 2.95 (95% CI: 2.05–4.25) for High CRD (*P* for trend < 0.001). Restricted cubic splines confirmed a non-linear association with accelerating risk at higher cCRDI scores (*P* for non-linearity = 0.008). Crucially, increasing CRD severity was independently associated with aggressive clinicopathological features, including multifocality, extrathyroidal extension (ETE), and a dramatic increase in lymph node metastasis (LNM) (69.4% in High CRD vs. 29.3% in No CRD, *P* < 0.001). High CRD remained the strongest independent predictor for LNM in multivariable analysis (aOR = 4.21, 95% CI: 2.90–6.10, *P* < 0.001).

**Conclusion:**

Cumulative CRD is independently associated with both the presence of malignancy and the aggressive progression of TC in patients with thyroid nodules. Assessing a patient’s circadian health might have potential utility as a novel, modifiable factor for risk stratification and may underscore the importance of lifestyle interventions in the clinical management of thyroid nodules.

## 1 Introduction

Thyroid nodules are a ubiquitous clinical finding, with a prevalence reaching up to 68% in the general population due to the widespread application of high-resolution ultrasonography (1). While the vast majority of these nodules are benign, approximately 5% to 15% harbor malignancy, predominantly papillary thyroid carcinoma (PTC) (2). The rapidly rising incidence of thyroid cancer (TC) globally has intensified the clinical challenge of distinguishing malignant from benign lesions, aiming to avoid both overtreatment of benign nodules and delayed intervention for aggressive carcinomas (3, 4). Currently, established risk factors for TC include advanced age, genetic predisposition [e.g., familial adenomatous polyposis (5)], history of childhood radiation exposure (6). However, these factors are largely non-modifiable. Consequently, identifying modifiable lifestyle factors that drive the malignant transformation of thyroid nodules is of paramount public health importance, as it could offer novel strategies for risk stratification and early preventive interventions.

In modern society, circadian rhythm disruption (CRD)—characterized by shift work, insufficient sleep duration, and delayed chronotypes—has emerged as a pervasive public health concern (7, 8). The endogenous circadian clock, governed by transcription-translation feedback loops involving core genes (e.g., CLOCK, BMAL1, PER, CRY), orchestrates 24-hour rhythms in cellular processes critical to genomic integrity, including cell cycle progression, DNA damage repair, apoptosis, metabolic homeostasis, and immune surveillance (9–11). Chronic misalignment of this biological clock has been robustly linked to oncogenesis; notably, the International Agency for Research on Cancer (IARC) has classified night shift work as a Group 2A “probable human carcinogen” (12). The proposed biological mechanisms linking CRD to cancer involve the suppression of nocturnal melatonin secretion, which serves as a potent antioxidant and oncostatic hormone, as well as systemic immune dysregulation and chronic low-grade inflammation (13, 14). While the carcinogenic effects of CRD have been well-documented in breast, prostate, and colorectal cancers, its specific impact on the endocrine system remains an area of active investigation (15–17). Recent studies have specifically linked short sleep duration and poor sleep quality to an increased prevalence of thyroid nodules and altered circadian gene expression in nodule patients (18–21).

The hypothalamic-pituitary-thyroid (HPT) axis exhibits a distinct circadian rhythm, with thyroid-stimulating hormone (TSH) levels typically peaking during the night (22, 23). CRD can significantly blunt this nocturnal TSH surge and perturb peripheral thyroid hormone metabolism. While this may appear counterintuitive in the context of TC risk, circadian misalignment could nonetheless foster a microenvironment conducive to malignant transformation through mechanisms beyond TSH alone (24, 25). Compelling experimental evidence further supports this link: BMAL1 knockout in thyroid cells decreases NFKBIA expression, accelerates cellular senescence, and impairs hormone synthesis, directly implicating circadian dysfunction in thyroid cellular decline (26). This is further supported by systematic reviews indicating that dysregulation of core circadian clock genes is a hallmark of malignant transformation in thyroid tissue (27), and that evaluating circadian disruption may offer novel insights into thyroid cancer etiology (28). Additionally, environmental disruptors inducing circadian misalignment (e.g., cortisone, TBPH) have been associated with thyroid endocrine disruption and elevated TC risk in preclinical models. Melatonin administration has demonstrated protective effects against circadian disruption-induced thyroid dysfunction and tumor progression, underscoring the therapeutic relevance of circadian pathways (29, 30).

Despite this mechanistic plausibility, epidemiological evidence regarding the association between CRD and TC is scarce and conflicting. Most prior studies compared TC patients with healthy controls (31, 32), failing to address the more pressing clinical dilemma: among patients already presenting with thyroid nodules, does CRD increase the likelihood of malignancy? Furthermore, many existing investigations rely on ultrasonographic diagnosis or fine-needle aspiration (FNA) cytology, which are susceptible to misclassification biases—particularly in indeterminate nodules (Bethesda III–V)—compared to the gold standard of postoperative histopathology (33). Molecular profiling has emerged to refine risk stratification in indeterminate nodules (34, 35), yet its integration with circadian metrics remains unexplored. Crucially, no study to date has rigorously evaluated CRD as an independent, modifiable risk factor for malignancy specifically within a histopathologically confirmed cohort of thyroid nodule patients.

To bridge this critical knowledge gap, we conducted a rigorous study utilizing a surgical cohort with definitive histopathological diagnoses. To minimize the confounding effects of baseline demographic disparities, we employed a propensity score matching (PSM) analysis. The primary objective of this study was to investigate the association between various dimensions of CRD—including shift work history, sleep duration, and chronotype—and the risk of malignancy in patients with thyroid nodules. We hypothesized that severe CRD is an independent risk factor for thyroid malignancy. By elucidating this relationship, these findings are intended to provide evidence-based lifestyle recommendations for the conservative management of patients with thyroid nodules.

## 2 Materials and methods

### 2.1 Study design and ethical considerations

This hospital-based retrospective cohort study was conducted at the Department of Thyroid Surgery, Binzhou Medical University Hospital, Binzhou, Shandong, China. We reviewed the medical records of consecutive patients who underwent initial thyroidectomy for thyroid nodules between January 2016 and December 2019. The study protocol adhered to the ethical guidelines of the Declaration of Helsinki and was approved by the Institutional Review Board (IRB) of Binzhou Medical University Hospital (Approval No. KYLL-067). Written informed consent was obtained from all participants prior to data collection. Patients unable to provide written informed consent were not included, which may have introduced selection bias.

### 2.2 Participants and eligibility criteria

We consecutively recruited adult patients admitted for thyroidectomy due to thyroid nodules. The decision for surgery was based on current clinical guidelines, including suspicious ultrasonographic features, compression symptoms, or indeterminate cytology.

Inclusion Criteria: (1) Age between 18 and 65 years; (2) Preoperative diagnosis of thyroid nodules by high-resolution ultrasonography; (3) Underwent total thyroidectomy or lobectomy with definitive postoperative histopathological reports.

Exclusion Criteria: (1) History of other malignancies or previous neck irradiation (to exclude radiation-induced carcinomas); (2) Diagnosis of severe psychiatric disorders (e.g., major depression, schizophrenia) or regular use of sedatives/hypnotics within the past 6 months (to eliminate pharmacological interference with sleep architecture); (3) Pregnant or lactating women; (4) Incomplete questionnaire data preventing the calculation of the cumulative circadian rhythm disruption index (cCRDI).

### 2.3 Data collection and covariates

Baseline demographic and clinical data were extracted from electronic medical records and face-to-face interviews conducted by trained research assistants 24 hours prior to surgery.

Demographic Variables: Age, sex, body mass index (BMI, calculated as kg/m²), and educational level.

Clinical Parameters: Thyroid function tests—including serum TSH and thyroid autoantibodies (TPOAb, TgAb) were measured using fasting blood samples collected on the morning of admission.

Ultrasonographic Features: Thyroid nodules were characterized according to the ACR TI-RADS classification (size, composition, echogenicity, shape, and margins).

### 2.4 Assessment of cCRDI

To comprehensively evaluate the cumulative impact of CRD on TC progression, we developed a novel metric: the cCRDI. The cCRDI was designed as an exploratory composite exposure index intended to summarize cumulative circadian burden, rather than to imply strict biological equivalence or identical effect sizes across its individual components. Conceptually analogous to the “pack-years” metric used in smoking history, the cCRDI integrates the intensity of circadian disruption with its duration of exposure. This index aims to quantify the long-term allostatic and metabolic burden imposed by adverse chronobiological behaviors on the endocrine and immune systems. Patients were instructed to report their habitual sleep, work, and dietary patterns during the 12-month period preceding the initial clinical or ultrasonographic diagnosis of the thyroid nodule that ultimately led to surgery, in order to standardize the exposure window.

The cCRDI was derived from preoperatively administered lifestyle questionnaires and constructed through a three-step algorithmic approach:

Step 1: Assessment of CRD Intensity (0—11points)

The baseline intensity of recent CRD was evaluated across four dimensions, reflecting both central and peripheral circadian clock disturbances. The Intensity Score was calculated as the sum of these four domains (A + B + C+ D):

Domain A: Sleep Insufficiency (Reflecting central clock disruption and cortisol dysregulation)

0 points: Sufficient sleep (average daily sleep ≥7.0 hours)

1 point: Mild deprivation (6.0—6.9 hours)

2 points: Moderate deprivation (5.0—5.9 hours)

3 points: Severe deprivation (<5.0 hours)

Domain B: Shift Work History (Reflecting external photoperiod disruption and melatonin suppression)

0 points: No night-shift history

1 point: Rare night shifts (<3 days/month)

2 points: Frequent night shifts (3–8 days/month)

3 points: Rotating or permanent night shifts (≥8 nights crossing midnight per month)

Domain C: Chronotype/Mid-Sleep Timing (Reflecting internal circadian phase preference)

0 points: Typical sleep onset before 23:30

1 point: Occasional late sleep onset (24:00–01:00, 1–2 times/week)

2 points: Habitual late chronotype (persistent sleep onset after 01:00)

3 points: Persistent sleep onset after 02:00 or clinically diagnosed delayed sleep-wake phase disorder

Domain D: Dietary Irregularity (Reflecting metabolic misalignment of peripheral clocks, e.g., liver and thyroid)

0 points: Regular daily meals, no nocturnal eating habit.

1 point: Occasional irregularity (skipping breakfast or consuming high-calorie meals after 22:00, 1—2 times/week).

2 points: Severe irregularity (skipping breakfast and/or nocturnal eating ≥3 times/week).

Patients maintaining strictly regular chronobiological habits across all domains received an Intensity Score of 0.

Step 2: Determination of Exposure Duration (Years)

Patients self-reported the duration (in years) of the aforementioned adverse behaviors prior to diagnosis. To standardize retrospective time-range estimations, categorical responses were converted into continuous numerical equivalents using representative midpoints:

Recent onset/<1 year → assigned 0.5 years

1–3 years → assigned 2.0 years

3–5 years → assigned 4.0 years

5–10 years → assigned 7.5 years

>10 years → assigned 12.0 years (A conservative cap applied to mitigate the undue influence of extreme outliers).

For patients with an Intensity Score of 0, the duration was inherently designated as 0.

Step 3: Calculation of the cCRDI

The final cCRDI was computed using a multiplicative model:

cCRDI = Intensity Score **×** Duration

Based on the distribution of cCRDI scores, patients were categorized into four groups: No CRD (Q1, cCRDI=0), Low CRD (Q2, cCRDI values ranging from the lowest positive value to the 33rd percentile of the non-zero distribution), Moderate CRD (Q3, cCRDI values between the 34th and 66th percentiles), and High CRD (Q4, cCRDI values above the 66th percentile).

To ensure data robustness and minimize bias, patients with missing data in two or more intensity domains or missing duration data were excluded from the analysis. For patients missing a single intensity domain, a conservative imputation strategy was applied by assigning a score of 0 to the missing variable, thereby preventing the overestimation of CRD risk.

### 2.5 Outcome assessment and patient grouping

The primary outcome of the study was the definitive postoperative histopathological diagnosis of the resected thyroid nodules. All surgical specimens were processed using standard formalin-fixed, paraffin-embedding techniques. To ensure objective and unbiased assessment, all slides were independently reviewed by two senior pathologists who were blinded to the patients’ clinical data and circadian rhythm profiles. Any diagnostic discrepancies were resolved by a third expert pathologist to reach a consensus.

For the purpose of comparative analysis, patients within the cohort were categorized into two distinct groups based on this primary outcome:

Malignant Group: This group comprised patients with a histopathological diagnosis of differentiated thyroid carcinoma, including but not limited to PTC and follicular thyroid carcinoma (FTC).

Benign Group: This group included patients with a diagnosis of a benign thyroid condition, such as nodular goiter, follicular adenoma, or Hashimoto’s thyroiditis (HT).

For all patients in the malignant group, tumors were staged according to the 8th edition of the American Joint Committee on Cancer (AJCC) TNM staging system.

### 2.6 Statistical analysis

Continuous variables were expressed as mean ± standard deviation (SD) or median (interquartile range, IQR) depending on normality, while categorical variables were presented as frequencies and percentages. Differences between groups were compared using the Student’s t-test, Mann-Whitney U test, or Chi-square test, as appropriate.

PSM: To minimize selection bias and balance baseline confounders between the malignant and benign groups, a 1:1 PSM analysis was performed. The propensity score for each patient was calculated using a multivariable logistic regression model that included a comprehensive set of potential confounders identified from clinical knowledge and baseline data. These variables were: age, sex, body mass index (BMI, as a categorical variable), education level, history of diabetes, preoperative TSH levels, and thyroid autoimmune status (defined by TPOAb/TgAb positivity or a histopathological diagnosis of HT). A nearest-neighbor matching algorithm with a caliper width of 0.02 was applied. The balance of covariates before and after matching was assessed using the Standardized Mean Difference (SMD), with an SMD < 0.10 indicating negligible imbalance.

Association Analysis: Conditional logistic regression models were utilized to estimate Odds Ratios (ORs) and 95% Confidence Intervals (CIs) for the association between cCRDI quartiles and thyroid malignancy. Model 1 was unadjusted and Model 2 was further adjusted for residual clinical variables not included in the matching algorithm, specifically nodule size and preoperative TSH levels. To visualize the potential non-linear dose-response relationship between continuous cCRDI scores and malignancy risk, Restricted Cubic Splines (RCS) with three knots were employed.

All statistical analyses were performed using R software (version 4.2.0) or SPSS (version 26.0). A two-sided P-value < 0.05 was considered statistically significant.

## 3 Results

### 3.1 Baseline characteristics of the total cohort (pre-matching)

A total of 2,541 patients with thyroid nodules who underwent surgical resection were included in this study. Based on definitive postoperative histopathology, the total cohort comprised 1,649 patients in the malignant group and 892 patients in the benign group. The baseline demographic and clinical characteristics of the unmatched cohort are detailed in **Table 1**.

Prior to matching, several significant differences were observed between the two groups, indicating the presence of confounding factors. Patients with thyroid malignancy were significantly younger than those with benign nodules (mean age: 40.5 ± 9.6 vs. 46.8 ± 11.5 years, *P* < 0.001), with a notably higher proportion of individuals aged under 55 years (80.0% vs. 70.0%, *P* < 0.001). There was also a marginal but statistically significant difference in BMI distribution (*P* = 0.045). Clinically, as expected for surgically resected specimens, malignant nodules were significantly smaller in diameter (1.08 ± 0.79 vs. 2.55 ± 1.40 cm, *P* < 0.001) and were associated with slightly higher preoperative TSH levels (1.90 vs. 1.80 mIU/L, *P* = 0.035).

Crucially, in the unmatched cohort, the distribution of CRD categories differed dramatically. The malignant group had a substantially higher proportion of patients with High CRD compared to the benign group (27.8% vs. 16.0%), whereas the benign group had more patients with No CRD (22.0% vs. 12.0%, *P* < 0.001 for trend).

### 3.2 PSM cohort

To rigorously control for the aforementioned baseline disparities and isolate the independent effect of circadian disruption, a 1:1 PSM was performed. This procedure successfully yielded a highly balanced matched cohort consisting of 850 pairs (850 malignant cases and 850 benign cases, total N = 1,700).

Following PSM, all incorporated covariates were optimally balanced between the two groups. As shown in **Table 1**, the SMDs for all matched variables—including age (SMD = 0.029), gender (SMD = 0.014), BMI category (SMD = 0.012), education level, diabetes, TSH levels, and thyroid autoimmune status (TPOAb, TgAb, and HT)—decreased to well below the stringent threshold of 0.10. Correspondingly, all *P*-values for these covariates exceeded 0.05, indicating negligible residual confounding. Subsequent primary analyses were conducted on this matched cohort to ensure robust causal inference.

**Table 1:** Baseline characteristics of the study population before and after PSM.

Variables	Before Matching (Total Cohort)				After Matching (PSM Cohort)			
	Benign Controls (n = 892)	Malignant Cases (n = 1,649)	*P* Value	SMD	Benign Controls (n = 850)	Malignant Cases (n = 850)	*P* Value	SMD
Age, years (Mean±SD)	46.8 ± 11.5	40.5 ± 9.6	<0.001	0.582	41.2 ± 10.5	40.9 ± 9.8	0.542	0.029
Age Group, n (%)			<0.001	0.235			0.765	0.015
< 55 years	624 (70.0)	1,319 (80.0)			680 (80.0)	685 (80.6)		
≥ 55 years	268 (30.0)	330 (20.0)			170 (20.0)	165 (19.4)		
Gender			0.215	0.052			0.785	0.014
Male	205 (23.0)	415 (25.2)			215 (25.3)	220 (25.9)		
Female	687 (77.0)	1,234 (74.8)			635 (74.7)	630 (74.1)		
BMI category			0.045	0.125			0.952	0.012
< 18.5	35 (3.9)	75 (4.5)			39 (4.6)	38 (4.5)		
18.5-22.9	380 (42.6)	730 (44.3)			375 (44.1)	376 (44.2)		
23.0-24.9	260 (29.1)	490 (29.7)			255 (30.0)	253 (29.8)		
≥ 25.0	217 (24.3)	354 (21.5)			181 (21.3)	183 (21.5)		
Education Level			0.052	0.080			0.895	0.008
High School or below	482 (54.0)	825 (50.0)			428 (50.4)	425 (50.0)		
College or above	410 (46.0)	824 (50.0)			422 (49.6)	425 (50.0)		
Diabetes	71 (8.0)	99 (6.0)	0.058	0.076	51 (6.0)	52 (6.1)	0.925	0.004
Nodule Size	2.55 ± 1.40	1.08 ± 0.79	<0.001	1.250	2.10 ± 1.15	1.05 ± 0.75	<0.001	1.100
TSH, mIU/L	1.80 [1.2-2.6]	1.90 [1.3-2.9]	0.035	0.085	1.85 [1.2-2.8]	1.88 [1.3-2.9]	0.412	0.025
TPOAb Positivity, n (%)	165 (18.5)	330 (20.0)	0.354	0.038	165 (19.4)	170 (20.0)	0.754	0.015
TgAb Positivity, n (%)	134 (15.0)	280 (17.0)	0.185	0.055	140 (16.5)	145 (17.1)	0.738	0.016
HT, n (%)	187 (21.0)	389 (23.6)	0.125	0.062	195 (22.9)	200 (23.5)	0.765	0.014
CRD Category, n (%)			<0.001				<0.001	
No CRD (Q1)	196 (22.0)	198 (12.0)			187 (22.0)	102 (12.0)		
Low CRD (Q2)	375 (42.0)	638 (38.7)			357 (42.0)	329 (38.7)		
Moderate CRD (Q3)	178 (20.0)	355 (21.5)			170 (20.0)	183 (21.5)		
High CRD (Q4)	143 (16.0)	458 (27.8)			136 (16.0)	236 (27.8)		

Data are presented as mean ± standard deviation (SD), median [interquartile range, IQR], or n (%). Propensity score matching (PSM) was performed on a 1:1 basis. The balance of covariates was assessed using the Standardized Mean Difference (SMD), with a value < 0.10 indicating a negligible imbalance. PSM, propensity score matching; SMD, standardized mean difference; BMI, body mass index; TSH, thyroid-stimulating hormone; TPOAb, thyroid peroxidase antibody; TgAb, thyroglobulin antibody; HT, Hashimoto's thyroiditis; CRD, circadian rhythm disruption.

### 3.3 Independent association between CRD and thyroid malignancy in the matched cohort

In the rigorously matched cohort, where baseline demographic, metabolic, and autoimmune confounders were successfully equalized, the significant association between the severity of CRD and the risk of thyroid malignancy persisted robustly (**Table 2**).

**Table 2:** Association between CRD and the risk of thyroid malignancy in the PSM cohort.

Variables	Benign Controls (n = 850)	Malignant Cases (n = 850)	Model 1		Model 2	
			OR (95% CI)	*P* Value	Adjusted OR (95% CI)	*P* Value
CRD Category, n (%)						
No CRD (Q1)	187 (22.0)	102 (12.0)	1.00 (Reference)		1.00 (Reference)	
Low CRD (Q2)	357 (42.0)	329 (38.7)	1.69 (1.25–2.28)	<0.001	1.58 (1.15–2.15)	0.004
Moderate CRD (Q3)	170 (20.0)	183 (21.5)	1.97 (1.41–2.75)	<0.001	1.85 (1.30–2.62)	<0.001
High CRD (Q4)	136 (16.0)	236 (27.8)	3.18 (2.28–4.44)	<0.001	2.95 (2.05–4.25)	<0.001
*P* for trend				<0.001		<0.001

Model 1: Unadjusted conditional logistic regression model based on the PSM (already inherently balanced for age, gender, BMI category, education level, diabetes, and HT).

Model 2: Multivariable conditional logistic regression model further adjusted for variables not included in the matching algorithm, specifically nodule size and preoperative TSH levels.

OR, odds ratio; CI, confidence interval; CRD, circadian rhythm disruption.

To quantify this risk, we performed multivariable conditional logistic regression analyses. Using patients with strict circadian regularity (No CRD, Q1) as the reference group, a clear, dose-dependent escalation in the risk of thyroid malignancy was observed across increasing CRD categories. In the unadjusted model within the PSM cohort (Model 1), the odds of harboring a malignancy increased progressively: patients in the Low, Moderate, and High CRD groups exhibited Odds Ratios (ORs) of 1.69 (95% CI: 1.25–2.28, *P* < 0.001), 1.97 (95% CI: 1.41–2.75, *P* < 0.001), and 3.18 (95% CI: 2.28–4.44, *P* < 0.001), respectively.

To ensure the utmost rigor, we constructed a fully adjusted model (Model 2) to account for residual clinical variables not included in the PSM algorithm, specifically preoperative TSH levels and nodule size (which inherently differ between benign and malignant pathologies). Even after this stringent adjustment, the independent detrimental effect of CRD remained highly significant. Compared to the No CRD group, the adjusted ORs for the Low, Moderate, and High CRD categories were 1.58 (95% CI: 1.15–2.15, *P* = 0.004), 1.85 (95% CI: 1.30–2.62, *P* < 0.001), and 2.95 (95% CI: 2.05–4.25, *P* < 0.001).

**Figure 1:**
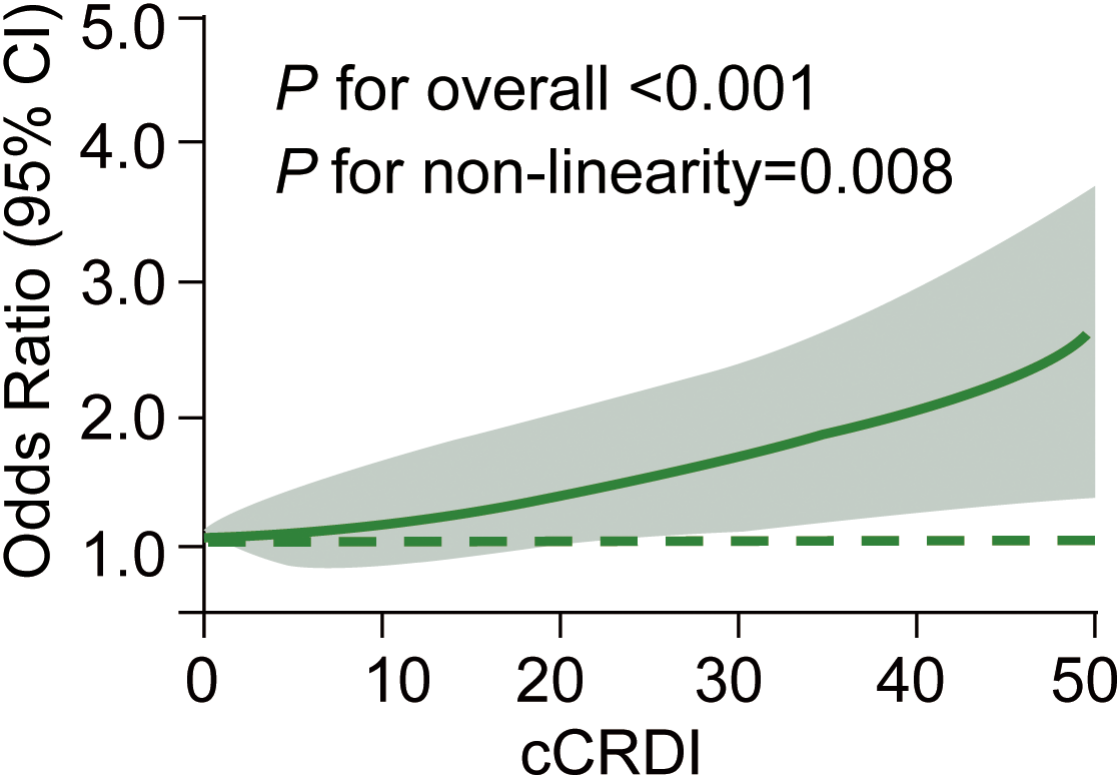
Non-linear dose-response relationship between the cCRDI and the risk of thyroid malignancy. the solid green line represents the adjusted or and the shaded gray area represents the 95% ci, derived from a restricted cubic spline model with three knots. the model was adjusted for nodule size and TSH levels. the dashed green line indicates the reference or of 1.0, with the reference point set at a cCRDI of 0. *p*-values for the overall association and for non-linearity are shown. cCRDI, cumulative circadian rhythm disruption index; OR, odds ratio; CI, confidence interval.

Crucially, the trend test across all quartiles in both models demonstrated a highly significant positive correlation (*P* for trend < 0.001). To further explore the shape of this association, we used restricted cubic splines to model the continuous cCRDI score. The analysis revealed a significant non-linear relationship (*P* for non-linearity = 0.008), characterized by a steep acceleration in risk at higher cCRDI values (**Figure 1**). In exploratory analyses, each individual component of the cCRDI—sleep insufficiency, shift work history, late chronotype, and dietary irregularity—was independently associated with an increased risk of malignancy in the matched cohort (**Supplementary Table 1**). This robust, non-linear dose-response relationship underscores that the cumulative burden of circadian disruption acts as a strong, independent driver of malignant transformation in patients presenting with thyroid nodules. Each component was analyzed in a separate conditional logistic regression model in the matched cohort, additionally adjusting for nodule size and preoperative TSH. In trend analyses treating each component score as an ordinal variable, every 1-point increase was significantly associated with malignancy.

### 3.4 Subgroup analyses: consistency across populations

Given that sleep patterns and chronotype are known to vary with age, we examined the correlation between cCRDI and age. In the matched cohort, cCRDI scores showed no significant correlation with age (Spearman’s ρ = 0.11, P = 0.07). To further ensure that age did not confound the primary association, we additionally adjusted for continuous age in the multivariable Model 2 (**Table 2**). The adjusted OR for High CRD remained substantially unchanged (AOR = 2.92, 95% CI: 2.01–4.24), confirming the independence of the observed effect. While this approach does not fully exclude age-related behavioral variation in individual cCRDI components, the absence of material change in the effect estimates suggests that the overall association between cCRDI and malignancy was not solely attributable to age differences.

**Figure 2:**
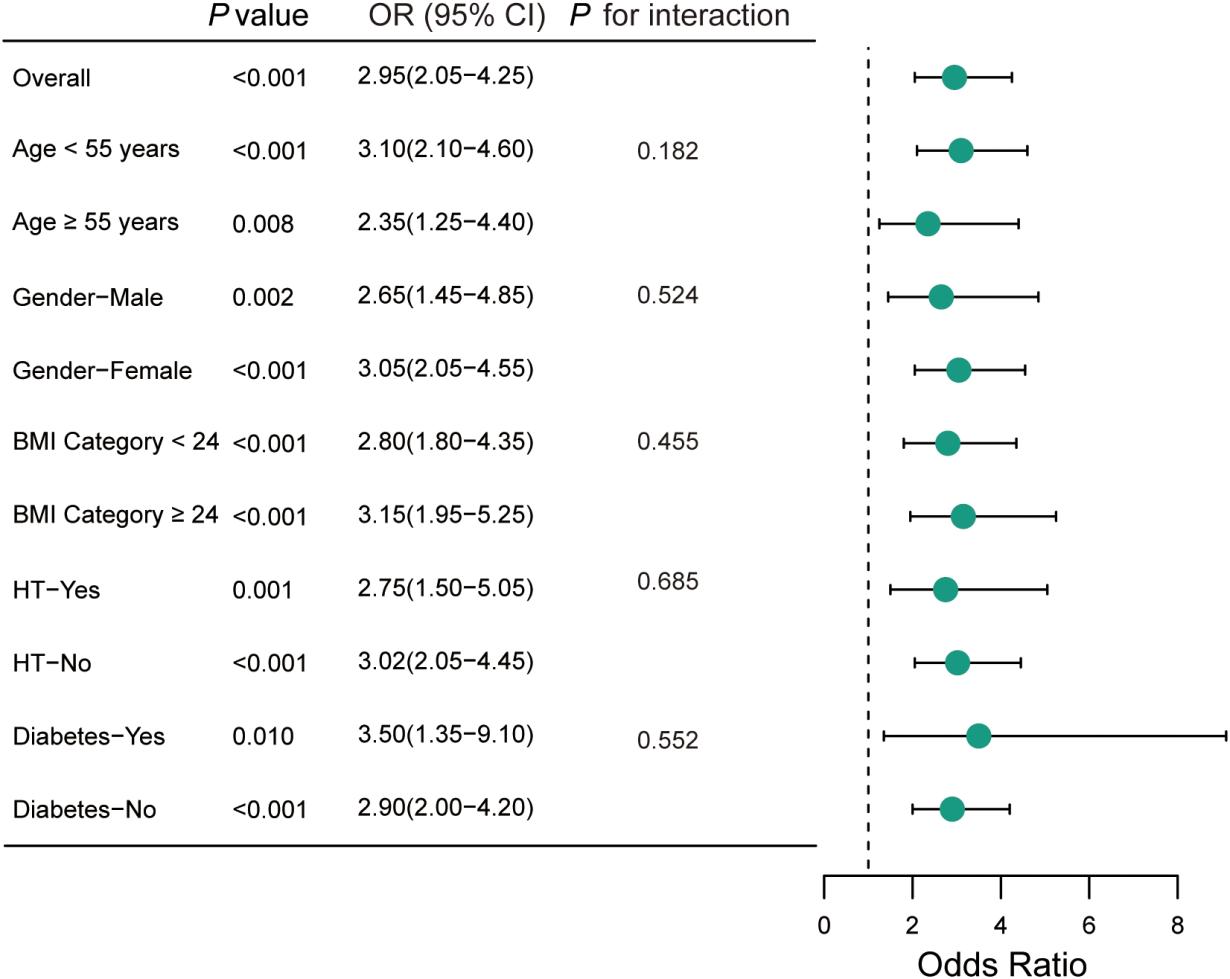
Subgroup analysis of the association between high CRD and thyroid malignancy risk. The forest plot displays the adjusted ORs and 95% CIs for the association between High CRD (Q4) versus No CRD (Q1) and the risk of thyroid malignancy across various subgroups. The analysis is based on the propensity score-matched cohort and adjusted for nodule size and TSH levels. The *P*-value for interaction was calculated to assess for effect modification by the subgroup variable. CRD, circadian rhythm disruption; OR, odds ratio; CI, confidence interval; BMI, body mass index; HT, Hashimoto’s thyroiditis.

To validate the consistency of our findings, stratified analyses were performed across various predefined demographic and clinical subgroups (**Figure 2**).

The detrimental impact of High CRD (vs. No CRD) on thyroid malignancy risk remained consistent and statistically significant across all strata. Notably, the risk magnitude appeared slightly more pronounced in younger patients (<55 years: OR = 3.10, 95% CI: 2.10–4.60) compared to older patients (≥55 years: OR = 2.35, 95% CI: 1.25–4.40), although the interaction term did not reach statistical significance (*P* for interaction = 0.182). Similarly, the association was observed in both males (OR = 2.65, 95% CI: 1.45–4.85) and females (OR = 3.05, 95% CI: 2.05–4.55), as well as in patients with and without HT. These findings suggest that the carcinogenic effect of CRD is a universal phenomenon among patients with thyroid nodules, independent of their baseline metabolic or autoimmune status.

### 3.5 Impact of CRD on clinicopathological aggressiveness in thyroid carcinoma

**Figure 3:**
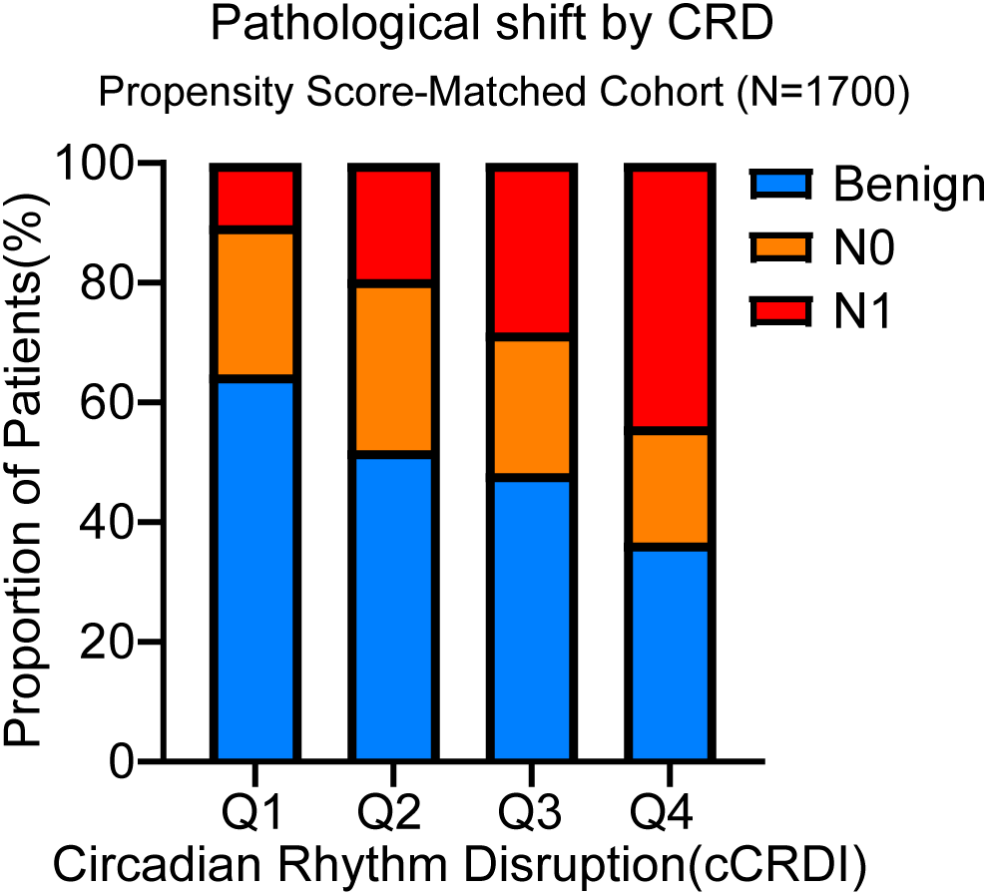
Proportional distribution of pathological outcomes across CRD categories. The 100% stacked bar chart illustrates the shift in the composition of pathological diagnoses across the four CRD categories within the propensity score-matched cohort (n=1,700). Each bar is segmented to show the proportion of patients with benign nodules, non-metastatic (N0) thyroid carcinoma, and metastatic (N1) thyroid carcinoma. CRD, circadian rhythm disruption; N0, no lymph node metastasis; N1, presence of lymph node metastasis.

Beyond its role in the initial malignant transformation, we first sought to visualize how CRD alters the overall landscape of pathological outcomes. A striking shift was observed across CRD categories: as CRD severity increased, the proportion of benign nodules dramatically decreased, while the proportion of thyroid carcinomas with LNM surged (**Figure 3**). This visual evidence suggests that CRD not only promotes carcinogenesis but also drives a more aggressive phenotype. To dissect this further, we analyzed detailed histopathological features within the malignant cohort (n = 1,649), stratified by CRD severity (**Table 3**).

**Table 3:** Association between CRD and clinicopathological aggressiveness in patients with TC (n = 1,649).

Variables	No CRD (n = 198)	Low CRD (n = 638)	Moderate CRD (n = 355)	High CRD (n = 458)	*P* Value
Tumor Size, cm (Mean ± SD)	0.97 ± 0.65	1.02 ± 0.72	1.09 ± 0.81	1.15 ± 0.88	0.012
Tumor Size Category, n (%)					0.009
≤ 1 cm	136 (68.7)	408 (64.0)	205 (57.7)	253 (55.2)	
> 1 cm	62 (31.3)	230 (36.0)	150 (42.3)	205 (44.8)	
Multifocality, n (%)					0.008
Unifocal	165 (83.3)	490 (76.8)	255 (71.8)	300 (65.5)	
Multifocal	33 (16.7)	148 (23.2)	100 (28.2)	158 (34.5)	
Bilaterality, n (%)					0.045
Unilateral	170 (85.9)	520 (81.5)	275 (77.5)	338 (73.8)	
Bilateral	25 (12.6)	108 (16.9)	75 (21.1)	110 (24.0)	
Isthmus only	3 (1.5)	10 (1.6)	5 (1.4)	10 (2.2)	
ETE, n (%)					0.012
Absent	178 (89.9)	557 (87.3)	298 (83.9)	370 (80.8)	
Present	20 (10.1)	81 (12.7)	57 (16.1)	88 (19.2)	
Vascular/Perineural Invasion, n (%)					0.042
Absent	197 (99.5)	628 (98.4)	345 (97.2)	440 (96.1)	
Present	1 (0.5)	10 (1.6)	10 (2.8)	18 (3.9)	
pT Stage, n (%)					0.010
T1 / T2	192 (97.0)	604 (94.7)	332 (93.5)	413 (90.2)	
T3 / T4	6 (3.0)	34 (5.3)	23 (6.5)	45 (9.8)	
pN Stage, n (%)					<0.001
N0 (No Metastasis)	140 (70.7)	380 (59.6)	160 (45.1)	140 (30.6)	
N1a (Central Compartment)	50 (25.3)	210 (32.9)	145 (40.8)	200 (43.7)	
N1b (Lateral Neck)	8 (4.0)	48 (7.5)	50 (14.1)	118 (25.8)	
Central LNM, n (%)					<0.001
Negative	140 (70.7)	380 (59.6)	160 (45.1)	140 (30.6)	
Positive	58 (29.3)	258 (40.4)	195 (54.9)	318 (69.4)	
Lateral LNM, n (%)					<0.001
Negative	190 (96.0)	590 (92.5)	305 (85.9)	340 (74.2)	
Positive	8 (4.0)	48 (7.5)	50 (14.1)	118 (25.8)	
Number of Positive LNM, Mean ± SD	1.20 ± 2.12	1.45 ± 2.56	2.60 ± 4.22	3.40 ± 5.80	<0.001
BRAF V600E Mutation, n (%)					0.780
Wild-type	20 (10.1)	60 (9.4)	30 (8.5)	35 (7.6)	
Mutant	30 (15.2)	96 (15.0)	59 (16.6)	84 (18.3)	
Unknown	148 (74.7)	482 (75.5)	266 (74.9)	339 (74.0)	

This analysis was performed on the entire cohort of patients with a malignant diagnosis. P-values were calculated to assess for trends or differences across the four CRD categories. SD, standard deviation; ETE, extrathyroidal extension; pT, pathological tumor stage; pN, pathological nodal stage; LNM, lymph node metastasis; BRAF, B-Raf proto-oncogene.

Strikingly, increasing severity of CRD was significantly associated with a stepwise escalation in tumor aggressiveness. Patients in the High CRD group presented with significantly larger primary tumors compared to the No CRD group (mean size: 1.15 ± 0.88 cm vs. 0.97 ± 0.65 cm, *P* = 0.012). Furthermore, severe circadian disruption was strongly correlated with a higher incidence of adverse pathological features, including multifocality (34.5% in High CRD vs. 16.7% in No CRD, *P* = 0.008), bilaterality (24.0% vs. 12.6%, *P* = 0.045), and ETE (19.2% vs. 10.1%, *P* = 0.012).

Most notably, CRD severity was a powerful predictor of lymphatic metastasis. The prevalence of central LNM (N1a) surged from 29.3% in patients with No CRD to a staggering 69.4% in those with High CRD (*P* < 0.001). Even more alarmingly, lateral LNM (N1b)—a hallmark of advanced disease requiring extensive neck dissection—was exceptionally rare in the No CRD group (4.0%) but highly prevalent in the High CRD group (25.8%, *P* < 0.001). The absolute number of metastatic lymph nodes also increased in a dose-dependent manner (mean 1.20 vs. 3.40 nodes, *P* < 0.001). These findings compellingly suggest that chronic circadian misalignment not only initiates oncogenesis but also actively promotes tumor invasion and metastatic dissemination in TC.

### 3.6 Independent predictors of LNM

To further dissect the mechanisms by which circadian disruption promotes a more aggressive phenotype, we performed a separate multivariable logistic regression analysis specifically within the malignant cohort (n = 1,649) to identify independent predictors for LNM (**Table 4**).

**Table 4:** Multivariable logistic regression analysis of risk factors for LNM in patients with TC (n = 1,649).

Variables	Univariate Analysis		Multivariable Analysis	
	OR (95% CI)	*P* Value	Adjusted OR (95% CI)	*P* Value
CRD Category				
No CRD (Q1)	1.00 (Reference)	–	1.00 (Reference)	–
Low CRD (Q2)	1.62 (1.20–2.19)	0.002	1.45 (1.05–2.00)	0.024
Moderate CRD (Q3)	2.50 (1.75–3.58)	<0.001	2.98 (2.12–4.18)	<0.001
High CRD (Q4)	5.45 (3.85–7.70)	<0.001	4.21 (2.90–6.10)	<0.001
*P* for trend		<0.001		<0.001
Age (< 55 vs. ≥ 55 years)	1.55 (1.20–2.00)	0.001	1.30 (1.05–1.60)	0.015
Gender (Male vs. Female)	1.25 (1.02–1.53)	0.032	1.15 (0.92–1.44)	0.215
Tumor Size (> 1 cm vs. ≤ 1 cm)	2.10 (1.75–2.52)	<0.001	1.85 (1.50–2.28)	<0.001
Multifocality (Yes vs. No)	1.70 (1.35–2.14)	<0.001	1.40 (1.10–1.78)	0.006
ETE (Yes vs. No)	2.25 (1.65–3.05)	<0.001	1.95 (1.40–2.70)	<0.001
HT (Yes vs. No)	1.28 (1.01–1.62)	0.041	1.10 (0.85–1.42)	0.455

The multivariable model included all variables listed in the table. The analysis was conducted on the entire cohort of patients with a malignant diagnosis to identify independent predictors for lymph node metastasis. LNM, lymph node metastasis; TC, thyroid cancer; OR, odds ratio; CI, confidence interval; AOR, adjusted odds ratio; ETE, extrathyroidal extension; HT, Hashimoto's thyroiditis; CRD, circadian rhythm disruption.

In the univariate analysis, High CRD demonstrated the strongest association with LNM among all examined variables, with a striking 5.45-fold increased risk compared to the No CRD group (OR = 5.45, 95% CI: 3.85–7.70, *P* < 0.001). Other significant predictors in the univariate setting included younger age (< 55 years), larger tumor size (> 1 cm), multifocality, and ETE.

After adjusting for this comprehensive set of conventional clinicopathological risk factors in the multivariable model, High CRD remained the most dominant and statistically significant independent predictor for LNM, with an adjusted odds ratio (AOR) of 4.21 (95% CI: 2.90–6.10, *P* < 0.001). This indicates that the observed increase in metastatic burden is not merely a secondary consequence of CRD-induced larger tumor size or more aggressive local invasion, but rather a direct biological consequence of circadian disruption itself. The risk of LNM also exhibited a clear dose-response relationship with CRD severity (*P* for trend < 0.001).

Younger age (< 55 years) also emerged as an independent risk factor for LNM (AOR = 1.30, 95% CI: 1.05–1.60, *P* = 0.015). In contrast, factors such as male gender and the presence of HT, which were significant in the univariate analysis, lost their statistical significance in the multivariable model, suggesting their association with LNM was likely confounded by other variables, primarily CRD.

## 4 Discussion

In this large, propensity score-matched study of a surgical cohort, we present emerging evidence that cumulative CRD is a potent and independent risk factor for both the presence and aggressiveness of malignancy in patients with thyroid nodules. Our primary finding reveals a striking dose-response relationship: the risk of a nodule being malignant escalates with increasing severity of CRD, with those in the highest quartile of our novel cCRDI exhibiting a nearly threefold increased risk compared to individuals with regular chronobiological habits. This association was confirmed to be non-linear, with a risk that accelerates sharply at higher levels of CRD, suggesting a potential biological threshold beyond which the body’s homeostatic mechanisms may be overwhelmed. Even more compellingly, our secondary analysis demonstrated that severe CRD is not merely an initiator of carcinogenesis but a powerful promoter of tumor progression. Increasing CRD severity was independently associated with a stepwise increase in adverse clinicopathological features, most notably a dramatic surge in the risk of both central and lateral LNM. These findings suggest CRD may be an important, modifiable lifestyle factor associated with the entire spectrum of TC development, from initial malignant transformation to metastatic dissemination.

Our study significantly advances the current understanding by addressing key limitations of prior epidemiological investigations. Previous studies, which have reported conflicting results, predominantly compared TC patients to healthy controls, failing to resolve the more pressing clinical dilemma of risk stratification among patients already presenting with thyroid nodules (31, 32). By utilizing a rigorously selected surgical cohort with definitive histopathological diagnoses, we eliminated the misclassification bias inherent in studies relying on ultrasonography or FNA cytology. Furthermore, the implementation of PSM successfully balanced a comprehensive set of demographic, metabolic, and autoimmune confounders, allowing for a more robust inference of CRD’s independent effect. Among the matched covariates, BMI warrants consideration because higher BMI has been associated with thyroid cancer risk in previous studies and may reflect underlying metabolic and inflammatory alterations (36, 37). The observed association between CRD and malignancy risk is biologically plausible, aligning with established knowledge of the HPT axis’s circadian regulation and the role of chronic TSH stimulation in promoting thyrocyte proliferation (22, 24, 25). While our multivariable model adjusted for TSH, the persistence of a strong association suggests that CRD’s oncogenic effects extend beyond simple HPT axis dysregulation, implicating other pathways such as systemic inflammation and immune dysfunction.

Perhaps the most striking and novel finding of our study is the profound impact of CRD on TC aggressiveness, particularly its role as the strongest independent predictor of LNM in our multivariable analysis (**Table 4**). This suggests that circadian misalignment creates a microenvironment conducive to invasion and metastasis. Several biological mechanisms likely underpin this phenomenon. Firstly, chronic CRD, especially through light-at-night exposure, suppresses nocturnal melatonin secretion, thereby depriving the body of a potent oncostatic agent known to inhibit tumor cell proliferation, invasion, and angiogenesis (13). Secondly, a misaligned central clock dysregulates the immune system, impairing the surveillance and elimination of metastatic cells by natural killer (NK) cells and cytotoxic T lymphocytes (9, 11). Thirdly, core clock genes such as BMAL1 directly regulate pathways critical to metastasis, including epithelial-mesenchymal transition (EMT) and cell adhesion. The impaired cellular function observed in BMAL1 knockout thyroid cells supports this direct mechanistic link (26). Notably, a systematic review found that among 12 tested circadian clock genes, 9 were significantly dysregulated in thyroid cancer tissue (27), and the expression levels of CLOCK and BMAL1 have been positively correlated with lymph node metastasis counts in thyroid carcinoma (38). The fact that CRD’s association with LNM remained robust after adjusting for tumor size and ETE suggests it may exert a direct pro-metastatic effect, a hypothesis that warrants urgent investigation in preclinical models. The observed association between younger age and LNM is consistent with prior studies showing that younger age is associated with a higher likelihood of lymph node metastasis or more extensive locoregional disease in papillary thyroid carcinoma, despite generally favorable overall survival (39, 40).

The clinical implications of our findings are suggestive but require prospective validation. The cCRDI, or a simplified version thereof, might eventually be integrated into clinical practice as a novel, non-invasive tool to aid in the risk stratification of thyroid nodules, particularly those with indeterminate cytology (Bethesda III-IV). A patient with a suspicious nodule and a high cCRDI score might warrant closer surveillance or a lower threshold for surgical intervention. For patients diagnosed with TC, a high cCRDI could potentially signal a higher *a priori* risk for occult metastases, which might influence the extent of the initial surgery, such as the decision to perform a prophylactic central neck dissection. From a public health perspective, our results provide preliminary evidence supporting the potential role of circadian health as a preventative strategy. For the vast population of patients with benign or low-risk thyroid nodules undergoing conservative management, lifestyle interventions aimed at regularizing sleep-wake cycles, optimizing meal timing, and avoiding night shift work may represent a crucial, patient-empowering strategy to potentially mitigate the risk of future malignant transformation.

This study has several notable strengths, including its large sample size, reliance on gold-standard histopathological outcomes, the development of a comprehensive cumulative exposure index (cCRDI), and the use of rigorous statistical methods to control for confounding. However, certain limitations must be acknowledged. Firstly, the design, although robust, is retrospective, making it susceptible to recall bias in the self-reported questionnaire data used to construct the cCRDI. While we have demonstrated a strong association, definitive causality can only be established through prospective studies. Secondly, as a single-center study, our findings may require validation in other populations with different genetic backgrounds and lifestyle patterns. Finally, the cCRDI, while innovative, is a subjective measure of chronodisruption. Future research should aim to corroborate these findings using objective markers such as actigraphy, serial melatonin or cortisol profiling, and clock gene expression analyses. Prospective cohort studies are imperative to confirm our findings and to investigate whether interventions aimed at restoring circadian rhythm can actively reduce the risk of malignancy and progression in patients with thyroid nodules.

Additionally, several specific limitations merit further discussion. First, the retrospective assessment of lifestyle behaviors during the 12-month period preceding the initial diagnosis of the index thyroid nodule is inherently vulnerable to differential recall bias, wherein patients with a cancer diagnosis may over-report adverse sleep and dietary habits, potentially inflating the observed associations. Specifically, the cCRDI captures a snapshot of behaviors during the 12-month period preceding the initial diagnosis of the index thyroid nodule and may not reflect lifetime exposure patterns. Therefore, an individual with a remote history of severe CRD (e.g., 20 years ago) who has recently adopted healthier habits would be misclassified into a lower CRD category, potentially biasing our results toward the null. Second, the cCRDI is a novel composite index that has not been externally validated; although exploratory analyses confirmed independent contributions of each component (**Supplementary Table 1**), the index requires validation against objective circadian biomarkers in future studies. Third, preoperative Bethesda cytology classifications were not systematically retrievable for this historical surgical cohort. We acknowledge this as an important limitation, because Bethesda category is a major determinant of baseline malignancy risk in patients with thyroid nodules and residual confounding by preoperative cytologic risk cannot be excluded. Nevertheless, the fact that no nodule in either group exceeded 4.0 cm suggests that resections performed solely for compressive symptoms were uncommon, partially mitigating concerns about confounding by surgical indication. However, the decision for surgery in this cohort was guided by standard clinical practice, including suspicious ultrasound features (ACR TI-RADS) and/or compressive symptoms, and the use of definitive histopathology as the outcome mitigates the risk of misclassification inherent in cytology-based studies. Finally, while we adjusted for ACR TI-RADS scores, residual confounding by unmeasured factors cannot be excluded, and definitive causal inference will require prospective cohort studies with objective circadian phenotyping.

## 5 Conclusion

In conclusion, this propensity score-matched study provides preliminary evidence that cumulative CRD is a potent, independent risk factor for malignancy in patients with thyroid nodules. This association follows a dose-response relationship, with a non-linear acceleration in risk at higher levels of chronodisruption, suggesting a threshold effect. Furthermore, beyond its role in carcinogenesis, CRD was independently associated with tumor aggressiveness, most notably LNM. These findings suggest the potential utility of assessing a patient’s circadian health as a novel, modifiable factor for both risk stratification and potential prevention in the management of thyroid nodules.

## Data Availability

The original contributions presented in the study are included in the article/**Supplementary Material**. Further inquiries can be directed to the corresponding authors.
