# Supplementary material for: Association between circadian rhythm disruption and the risk of malignancy in patients with thyroid nodules: a propensity score-matched study
Source: Front Endocrinol (Lausanne). 2026 May 12;17:1832788. doi: 10.3389/fendo.2026.1832788 (PMC13201181; doi:10.3389/fendo.2026.1832788)
Supplement: Supplementary file 1 [file Table1.docx]

Table S1. Adjusted Associations of Individual cCRDI Components with Thyroid Malignancy in the PSM Cohort

| **cCRDI Component** | **n (%) in Benign Controls (n=850)** | **n (%) in Malignant Cases (n=850)** | **Adjusted OR (95% CI) *** | **P Value** |
| --- | --- | --- | --- | --- |
| Sleep Insufficiency Score |  |  |  |  |
| 0 (Sufficient, ≥7h) | 365 (42.9) | 280 (32.9) | 1.00 (Reference) |  |
| 1 (Mild, 6.0–6.9h) | 290 (34.1) | 305 (35.9) | 1.32 (1.05–1.66) | 0.018 |
| 2 (Moderate, 5.0–5.9h) | 140 (16.5) | 170 (20.0) | 1.58 (1.20–2.09) | 0.001 |
| 3 (Severe, <5h) | 55 (6.5) | 95 (11.2) | 2.15 (1.48–3.12) | <0.001 |
| *Per 1-point increase* |  |  | 1.28 (1.15–1.43) | <0.001 |
| Shift Work Score |  |  |  |  |
| 0 (No night shifts) | 620 (72.9) | 540 (63.5) | 1.00 (Reference) |  |
| 1 (<3 nights/month) | 140 (16.5) | 165 (19.4) | 1.35 (1.04–1.75) | 0.023 |
| 2 (3–8 nights/month) | 60 (7.1) | 90 (10.6) | 1.72 (1.20–2.46) | 0.003 |
| 3 (≥8 nights/month) | 30 (3.5) | 55 (6.5) | 2.10 (1.31–3.37) | 0.002 |
| *Per 1-point increase* |  |  | 1.26 (1.12–1.42) | <0.001 |
| Late Chronotype Score |  |  |  |  |
| 0 (Sleep onset <23:30) | 430 (50.6) | 350 (41.2) | 1.00 (Reference) |  |
| 1 (Occasional late) | 250 (29.4) | 275 (32.4) | 1.35 (1.08–1.69) | 0.009 |
| 2 (Habitual >01:00) | 120 (14.1) | 150 (17.6) | 1.54 (1.16–2.05) | 0.003 |
| 3 (>02:00 or DSWPD) | 50 (5.9) | 75 (8.8) | 1.84 (1.25–2.72) | 0.002 |
| *Per 1-point increase* |  |  | 1.22 (1.10–1.35) | <0.001 |
| Dietary Irregularity Score |  |  |  |  |
| 0 (Regular meals) | 380 (44.7) | 305 (35.9) | 1.00 (Reference) |  |
| 1 (Occasional irregular) | 340 (40.0) | 360 (42.4) | 1.32 (1.07–1.63) | 0.011 |
| 2 (Severe irregular) | 130 (15.3) | 185 (21.8) | 1.77 (1.35–2.33) | <0.001 |
| *Per 1-point increase* |  |  | 1.31 (1.16–1.48) | <0.001 |

* Adjusted for nodule size and preoperative TSH levels using conditional logistic regression in the propensity score-matched cohort (850 benign vs. 850 malignant cases).Each component was entered into a separate conditional logistic regression model.Models were additionally adjusted for nodule size and preoperative TSH
Abbreviations: OR, odds ratio; CI, confidence interval; cCRDI, cumulative circadian rhythm disruption index; DSWPD, delayed sleep-wake phase disorder.
